# Ciprofloxacin has antifibrotic effects in scleroderma fibroblasts via downregulation of Dnmt1 and upregulation of Fli1

**DOI:** 10.3892/ijmm.2012.1150

**Published:** 2012-10-05

**Authors:** ANDREEA M. BUJOR, PAUL HAINES, CRISTINA PADILLA, ROMY B. CHRISTMANN, MONICA JUNIE, PERCIVAL D. SAMPAIO-BARROS, ROBERT LAFYATIS, MARIA TROJANOWSKA

**Affiliations:** 1 Arthritis Center, Rheumatology, Boston University School of Medicine, Boston, MA 02118, USA;; 2 Division of Microbiology and Immunology, Iuliu Hatieganu Medical University, Cluj Napoca 400012, Romania;; 3 Division of Rheumatology, University of São Paulo School of Medicine, Cerqueira César, São Paulo 01246903, Brazil

**Keywords:** systemic sclerosis, fibrosis, ciprofloxacin, collagen, matrix metalloproteinase 1, friend leukemia integration factor 1, DNA methyltransferase 1

## Abstract

Systemic sclerosis (SSc) is characterized by fibrosis of the skin and internal organs. The present study was undertaken to examine the effects of ciprofloxacin, a fluoroquinolone antibiotic implicated in matrix remodeling, on dermal and lung fibroblasts obtained from SSc patients. Dermal and lung fibroblasts from SSc patients and healthy subjects were treated with ciprofloxacin. Western blotting was used to analyze protein levels and RT-PCR was used to measure mRNA expression. The pharmacologic inhibitor UO126 was used to block Erk1/2 signaling. SSc dermal fibroblasts demonstrated a significant decrease in collagen type I mRNA and protein levels after antibiotic treatment, while healthy dermal fibroblasts were less sensitive to ciprofloxacin, downregulating collagen only at the protein levels. Connective tissue growth factor (CCN2) gene expression was significantly reduced and matrix metalloproteinase 1 (MMP1) levels were enhanced after ciprofloxacin treatment to a similar extent in healthy and SSc fibroblasts. Ciprofloxacin induced Erk1/2 phosphorylation, and Erk1/2 blockade completely prevented MMP1 upregulation. However, Smad1 and Smad3 activation in response to TGFβ was not affected. The expression of friend leukemia integration factor 1 (Fli1), a transcriptional repressor of collagen, was increased after treatment with ciprofloxacin only in SSc fibroblasts, and this was accompanied by a decrease in the levels of DNA methyltransferase 1 (Dnmt1). Similar effects were observed in SSc-interstitial lung disease (ILD) lung fibroblasts. In summary, our study demonstrates that ciprofloxacin has antifibrotic actions in SSc dermal and lung fibroblasts via the downregulation of Dnmt1, the upregulation of Fli1 and induction of MMP1 gene expression via an Erk1/2-dependent mechanism. Thus, our data suggest that ciprofloxacin may be an attractive therapy for SSc skin and lung fibrosis.

## Introduction

Systemic sclerosis (SSc) is a disease characterized by vasculopathy, activation of the immune system and exaggerated deposition of extracellular matrix (ECM), resulting in stiff skin and fibrosis of internal organs. Skin fibrosis in scleroderma may be dramatic, severely affecting the mobility and quality of life of a patient, while lung complications are responsible for the majority of deaths. Several therapeutic options are available for SSc patients, including immunomodulatory agents, but their efficacy in reversing fibrosis is controversial, being considered at most only effective in halting the progression of the skin and lung disease. Therefore, the development of alternative therapeutic agents is warranted.

Ciprofloxacin is a broad-spectrum antibiotic of the fluoroquinolone class that targets bacterial DNA gyrase, with satisfactory tissue distribution (1). Previous *in vivo* studies in animal models of fibrosis have suggested an antifibrotic role for ciprofloxacin. Thus, ciprofloxacin treatment significantly decreased hepatic fibrogenesis in bile duct ligated and carbon tetrachloride/ethanol cirrhotic rats (2,3). Furthermore, topical ciprofloxacin increased the incidence of corneal perforations, significantly delaying corneal wound healing (4,5) and in a separate study prolonged tympanic membrane perforation healing (6). Increased matrix metalloproteinase (MMP) synthesis in response to ciprofloxacin treatment has been reported in several cell types, including tenocytes (7–10), chondrocytes (11), corneal epithelial cells and corneal stromal keratocytes (4).

A recent double blind randomized clinical trial compared changes in skin fibrosis in placebo and ciprofloxacin-treated scleroderma patients. Using the modified Rodnan skin score (MRSS), investigators demonstrated that after six months of treatment there was a significant decrease in MRSS in patients treated with ciprofloxacin when compared with the placebo-treated group (58 vs. 18%). Importantly, no significant side effects were reported, suggesting that long-term use of this drug may be safe in SSc patients (12). While this report suggests that ciprofloxacin has antifibrotic effects on SSc skin, the mechanism of action in dermal fibroblasts is completely unknown.

Friend leukemia integration factor 1 (Fli1) is a member of the Ets family of transcription factors that is preferentially expressed in hematopoietic cell lineages (13). Although expressed at low levels in dermal fibroblasts, Fli1 plays a pivotal role in the regulation of ECM genes, including type I collagen (14–16) and the profibrotic matrix protein connective tissue growth factor (CCN2) (17). Fli1 is a potent inhibitor of collagen gene expression in dermal fibroblasts and the downregulation of Fli1 protein in dermal fibroblasts from the affected skin of SSc patients correlates with elevated collagen deposition, thus suggesting a role of Fli-1 in SSc fibrosis (15).

The present study was undertaken to examine the effects of ciprofloxacin on cultured human dermal fibroblasts from SSc patients and the normal controls. We demonstrate that ciprofloxacin reduces the expression of the fibrotic markers collagen type I, CCN2 and cartilage oligomeric matrix protein (COMP) and that it upregulates matrix metalloproteinase 1 (MMP1) gene expression via an Erk1/2 dependent mechanism. Our study also provides evidence that SSc fibroblasts are more sensitive to the antifibrotic effects of ciprofloxacin, presumably via DNA methyltransferase 1 (Dnmt1)-induced derepression of Fli1. Additionally, we demonstrate that ciprofloxacin has potent antifibrotic effects on lung fibroblasts isolated from SSc patients with interstitial lung disease (ILD).

## Materials and methods

### Reagents

The following antibodies were used: monoclonal β-actin (Sigma-Aldrich, St. Louis, MO, USA), anti-CTGF, anti-lamin A/C (Santa Cruz Biotechnology, Inc., Santa Cruz, CA, USA), goat anti-type-1 collagen (Southern Biotech, Birmingham, AL, USA), anti-phospho-ERK1/2 (T202/Y204), anti-ERK1/2, anti-SMAD2/3, anti-phospho-SMAD2/3 (S465/467), anti-SMAD1/5/8, anti-phospho-SMAD1/5 (S463/465)/SMAD8 (S426/428), monoclonal anti-Dnmt1 (Cell Signaling Technology, Inc., Beverly, MA, USA) and monoclonal anti-MMP1 (Millipore, Billerica, MA, USA). Polyclonal rabbit anti-Fli-1 was purchased from Aviva Systems Biology (Cat #: ARP38096_T020; San Diego, CA, USA). Ciprofloxacin was purchased from LKT Laboratories (St. Paul, MN, USA). Dulbecco’s modified Eagle’s medium (DMEM) and 100X antibiotic-antimycotic solution (penicillin streptomycin and amphotericin B) were obtained from Gibco-BRL (Grand Island, NY, USA). Fetal bovine serum (FBS) was purchased from HyClone (Logan, UT, USA). The ERK1/2 inhibitor UO126 was purchased from Cell Signaling Technology, Inc. Enhanced chemiluminescence reagent and bovine serum albumin (BSA) protein assay reagent were obtained from Pierce Biotechnology, Inc. (Rockford, IL, USA). Tri reagent was purchased from the Molecular Research Center (Cincinnati, OH, USA). Primers were purchased from Operon (Huntsville, AL, USA).

### Cell culture

Human dermal fibroblast cultures were established from biopsy specimens obtained from the dorsal forearms of SSc patients with diffuse cutaneous disease and from age-, race- and gender-matched healthy donors, upon informed consent and in compliance with the Institutional Review Board. Dermal fibroblasts were cultured from the biopsy specimens as previously described (24). Normal and SSc skin fibroblasts were cultured in DMEM supplemented with 10% FBS and 1% antibiotic-antimycotic solution. Primary lung fibroblast cell cultures were developed using the explant method immediately after open lung biopsy, upon informed consent and in compliance with the Institutional Review Board. The lung specimens were sectioned in small fragments and transferred to a large 25-cm^2^ flask (Costar, USA) containing DMEM supplemented with 20% FBS, 1% L-glutamine and 1% penicillin + streptomycin. The specimens were incubated at 37°C in a 5% CO_2_ atmosphere until cells achieved confluence. After cell detachment with 0.2% trypsin in phosphate-buffered saline (PBS), the cells were transferred to a 75-cm^2^ flask and grown in DMEM with 20% FBS.

Fibroblasts were grown to confluence, changed to serum-free media and treated with indicated doses of ciprofloxacin or 0.1 M HCI for 24, 48 or 96 h before mRNA or protein was collected. For the inhibition of ERK1/2, UO126 was applied (1 *μ*M) in the serum-free media 1 h prior to the beginning of ciprofloxacin treatment and the cells were harvested after 48 h.

### Western blot analysis

Cells were collected and washed with PBS. Cell pellets were suspended in lysis buffer containing 20 mM Tris-HCl, pH 7.5, 15 mM NaCl, 1 mM EDTA, 1 mM EGTA, 1% Triton X-100, 2.5 mM sodium pyrophosphate and 1 mM glycerophosphate with freshly added phosphatase inhibitors (5 mM sodium fluoride and 1 mM Na_3_VO_4_) and a protease inhibitor mixture (Sigma-Aldrich). Protein concentration was quantified using the BCA Protein assay kit (Thermo Scientific, Rockford, IL, USA). Equal amounts of total protein for each sample were separated via SDS-PAGE and transferred to nitrocellulose membranes (Bio-Rad, Hercules, CA, USA). Membranes were blocked in 2% milk in TBST for 1 h and incubated with the primary Ab overnight at 4°C. After TBST washes, membranes were probed with HRP-conjugated secondary Ab against the appropriate species for 1 h at room temperature. Protein was visualized using an ECL reagent (Amersham Biosciences, Piscataway, NJ, USA) and quantified using Image J densitometry software. For nuclear extraction, the NE-PER nuclear and cytoplasmic extraction kit was used according to the manufacturer’s instructions. For the loading controls, blots were stripped with Restore Western Blot Stripping buffer (all were from Thermo Scientific) and reprobed with antibodies to β-actin or lamin A/C, for whole cell or nuclear lysates, respectively.

### Real-time PCR

Total RNA was isolated from dermal fibroblasts using Tri reagent according to the manufacturer’s instructions. RNA (1 *μ*g) was reverse transcribed in a 20-*μ*l reaction using random primers and Transcriptor First Strand Synthesis kit (Roche Applied Science, Indianapolis, IN, USA). cDNA was diluted 10-fold and quantitative (q)PCR was performed using IQ SYBR Green mix on an iCycler PCR machine (all were from Bio-Rad) using 1 *μ*l of cDNA in triplicate, with β-actin as the internal control. The primers used were as follows: β2-microglobulin, forward (GCC GTG TGA ACC ATG TGA CTT T) and reverse (CCA AAT GCG GCA TCT TCA AA); MMP1, forward (TCT GGG GTG TGG TGT CTA) and reverse (GCC TCC CAT CAT TCT CAG GTT); COL1A1, forward (CCA GAA GAA CTG GTA CAT CAG CA) and reverse (CGC CAT ACT CGA ACT GGG AAT); COL1A2, forward (GAT GTT GAA CTT GTT GCT GAG G) and reverse (TCT TTC CCC ATT CAT TTG TCT T); Fli-1, forward (GGA TGG CAA GGA ACT GTG TAA) and reverse (GGT TGT ATA GGC CAG CAG); COMP, forward (GCA CCG ACG TCA ACG AGT) and reverse (TGG TGT TGA TAC AGC GGA CT); CCN2, forward (TTG CGA AGC TGA CCT GGA AGA GAA) and reverse (AGC TCG GTA TGT CTT CAT GCT GGT).

## Results

### Increased sensitivity of SSc fibroblasts to the antifibrotic effects of ciprofloxacin

**Figure 1:**
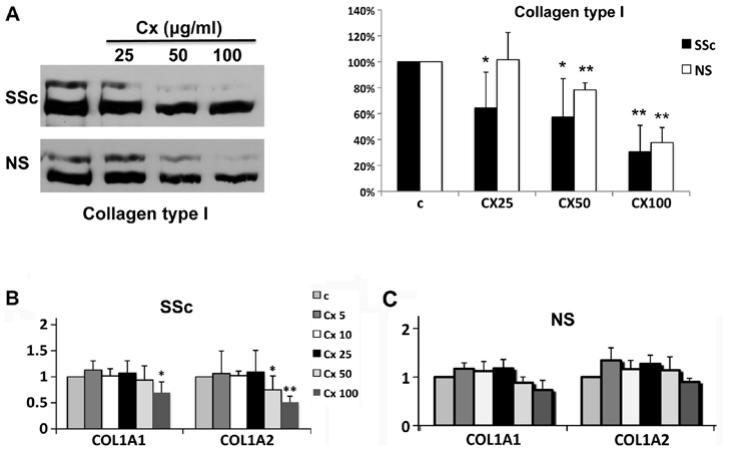
Systemic sclerosis dermal fibroblasts are more sensitive to ciprofloxacin compared to the control cells. Equal numbers of human dermal fibroblasts were plated, grown to confluence then serum-starved and treated with increasing doses of ciprofloxacin. Representative western blot analysis from conditioned media collected 48 h later is displayed in (A) with relative quantification in the graph bars at right. (B and C) RT-PCR analysis of mRNA levels of COL1A1 and COL1A2 are demonstrated, for systemic sclerosis and healthy cells. Bar graphs represent the means ± SEM of independent experiments in 5 systemic sclerosis and 5 healthy cell lines. ^*^P≤0.05 and ^**^P≤0.01. Cx, ciprofloxacin; NS, healthy cells; SSc, systemic sclerosis.

In order to compare the effects of ciprofloxacin on SSc and normal dermal fibroblasts, quiescent SSc and control cells were treated with increasing concentrations of ciprofloxacin, and collagen protein levels were analyzed in the culture supernatants. Ciprofloxacin more potently decreased collagen type I secretion in SSc cells compared to normal fibroblasts (Fig. 1A). Thus, there was a statistically significant decrease in secreted collagen starting with 25 *μ*g/ml of ciprofloxacin (∼40%, right panel), while the same dose had no effect on collagen type I production in normal control fibroblasts. However, at the highest dose tested (100 *μ*g/ml), ciprofloxacin potently downregulated collagen type I protein (>60%) in both normal and SSc dermal fibroblasts. Ciprofloxacin also inhibited mRNA expression of COL1A1 and COL1A2, displaying a similar dose response (Fig. 1B and C). Comparable to the results at the protein levels, there was a statistically significant decrease in both collagen type I gene expression in SSc cells, with COL1A2 being more responsive to ciprofloxacin treatment (Fig. 1B). Ciprofloxacin only modestly inhibited COL1A1 and COL1A2 mRNA expression by normal dermal fibroblasts, not reaching statistical significance (Fig. 1C). These data suggest that SSc dermal fibroblasts are more sensitive to the antifibrotic effects of ciprofloxacin compared to the healthy dermal fibroblasts.

### Ciprof loxacin downregulates CCN2 and COMP and increases MMP1 in human dermal fibroblasts

**Figure 2:**
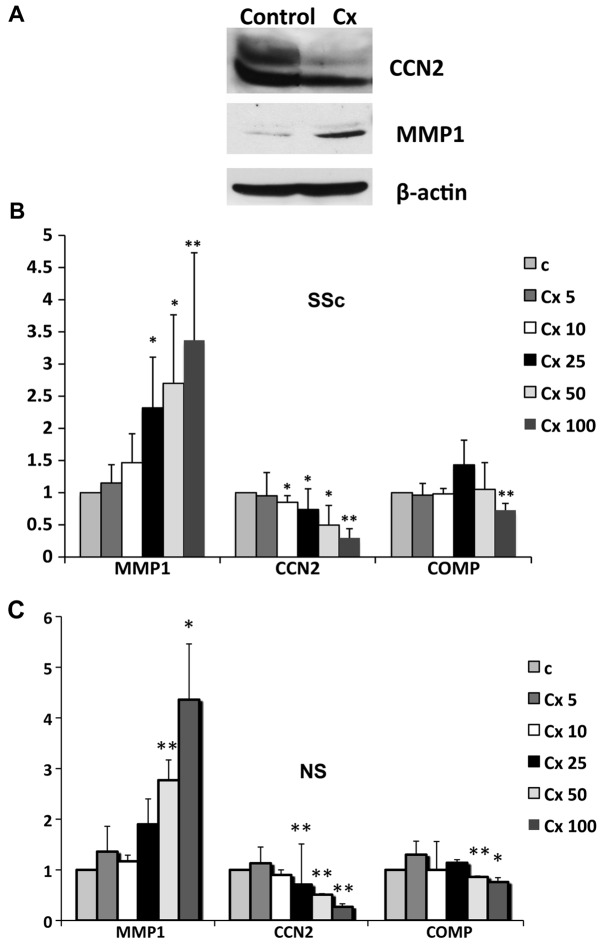
Ciprofloxacin induces MMP1 and inhibits CCN2 and COMP gene expression. Quiescent, serum-starved fibroblasts were treated (A) with 50 *μ*g/ml or (B and C) increasing doses of ciprofloxacin for 48 h. (A) Levels of MMP1 and CCN2 were analyzed by western blot analysis in cell layers and β-actin was used as a control for loading. (B and C) mRNA levels of MMP1, CCN2 and COMP were analyzed by RT-PCR in 5 different cell lines and the results are presented as bar graphs for systemic sclerosis and healthy cells. ^*^P≤0.05 and ^**^P≤0.01. Cx, ciprofloxacin; NS, healthy cells; SSc, systemic sclerosis.

We next examined the effects of ciprofloxacin treatment on other fibrotic markers that have been implicated in SSc pathogenesis. Protein levels of the profibrotic gene CCN2 and of matrix degrading metalloproteinase MMP1 were analyzed in cell lysates after antibiotic treatment. As previously reported in other cell types, ciprofloxacin increased MMP1 production in human dermal fibroblasts. Additionally, there was a significant decrease in CCN2 protein levels after antibiotic treatment, an effect that has not been previously described in any other cell types (Fig. 2A). To further investigate potential differences in response to ciprofloxacin treatment between SSc and healthy cells, we analyzed mRNA levels of MMP1, CCN2 and the cartilage oligomeric matrix protein (COMP). MMP1 gene expression was induced in a dose-dependent manner in both SSc and normal cells, starting with the smallest dose tested and to a similar extent in both cell types. Opposite effects were observed for CCN2 and COMP, which were decreased at the mRNA levels in both SSc and normal cells (Fig. 2B and C).

### Ciprofloxacin induces MMP1 gene expression via an Erk1/2-dependent mechanism

**Figure 3:**
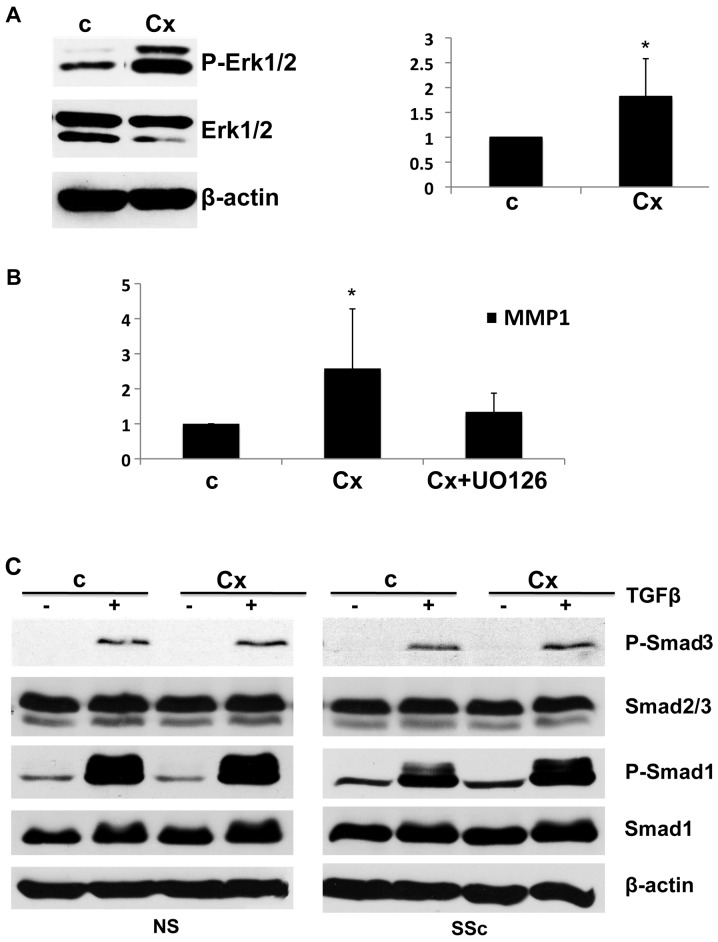
Ciprofloxacin induces Erk1/2 activation and has no effect on TGFβ/Smad signaling. (A) Cells were treated with 50 *μ*g/ml of ciprofloxacin and P-Erk1/2 levels were analyzed after 12 h; bar graph on the right represents relative quantification of 4 experiments. (B) Cells were pretreated with Erk inhibitor UO126 and mRNA levels of MMP1 were analyzed after 48 h of ciprofloxacin treatment (50 *μ*g/ml) (n=3). ^*^P≤0.05 and ^**^P≤0.01. (C) Healthy cells and systemic sclerosis dermal fibroblasts were pretreated with 50 *μ*g/ml of ciprofloxacin overnight then with 2.5 ng/ml of TGFβ and levels of P-Smad3, total Smad2/3, P-Smad1 and total Smad1 were analyzed by western blot analysis. Representative blots from 3 experiments are shown. Cx, ciprofloxacin; NS, healthy cells; SSc, systemic sclerosis; c, control.

Previous studies have demonstrated that in human dermal fibroblasts MMP1 gene expression is mainly controlled via an Erk1/2-dependent mechanism (18–23). To investigate the mechanism of ciprofloxacin-induced MMP1 gene upregulation we examined the effect of ciprofloxacin on phosphorylated Erk1/2 (P-Erk1/2). Ciprofloxacin significantly induced Erk1/2 phosphorylation in human dermal fibroblasts, with the levels of P-Erk1/2 almost doubling (Fig. 3A, right panel), suggesting that Erk1/2 may be involved in ciprofloxacin-induced MMP1 gene expression. To further confirm this we pretreated cells with a pharmacologic inhibitor of Erk1/2 (UO126) before ciprofloxacin treatment. Pretreatment with Erk1/2 inhibitor completely abolished ciprofloxacin-induced MMP1 upregulation (Fig. 3B). Together, these findings strongly support the notion that the activation of the Erk1/2 pathway contributes to ciprofloxacin-induced MMP1 upregulation in human dermal fibroblasts.

### Effects of ciprofloxacin on signaling pathways deregulated in SSc

Aberrant activation of several signaling pathways implicated in the pathogenesis of SSc may serve as a target for ciprofloxacin treatment. These include the upregulation of the major profibrotic TGFβ pathway, as well as the PI3K/Akt and PKCδ/c-abl/Fli1 pathways (24,25). TGFβ signaling plays a central role in SSc pathogenesis and is evidenced by an increased expression of pSmad1 in SSc skin and cultured fibroblasts (26), elevated phosphorylated Smad2/3 levels and increased nuclear localization of phosphorylated Smad2/3 in these cells (27).

To investigate the mechanism behind the antifibrotic actions of ciprofloxacin we evaluated its effects on TGFβ signaling. Pretreatment with ciprofloxacin had no effect on TGFβ-induced phosphorylation of Smad3 or Smad1 in both SSc and healthy cells, suggesting that the antifibrotic effects of ciprofloxacin are not mediated through the TGFβ/Smad pathway (Fig. 3C).

Akt activation has been previously linked to the regulation of collagen, MMP1 and CCN2 gene expression (19,28) and constitutive Akt activation has been reported in SSc fibroblasts (24). Similar to the results in TGFβ/Smad activation, ciprofloxacin treatment had no effects on Akt phosphorylation, suggesting that this pathway is not involved (data not shown).

### Ciprofloxacin increases Fli1 levels in SSc fibroblasts

**Figure 4:**
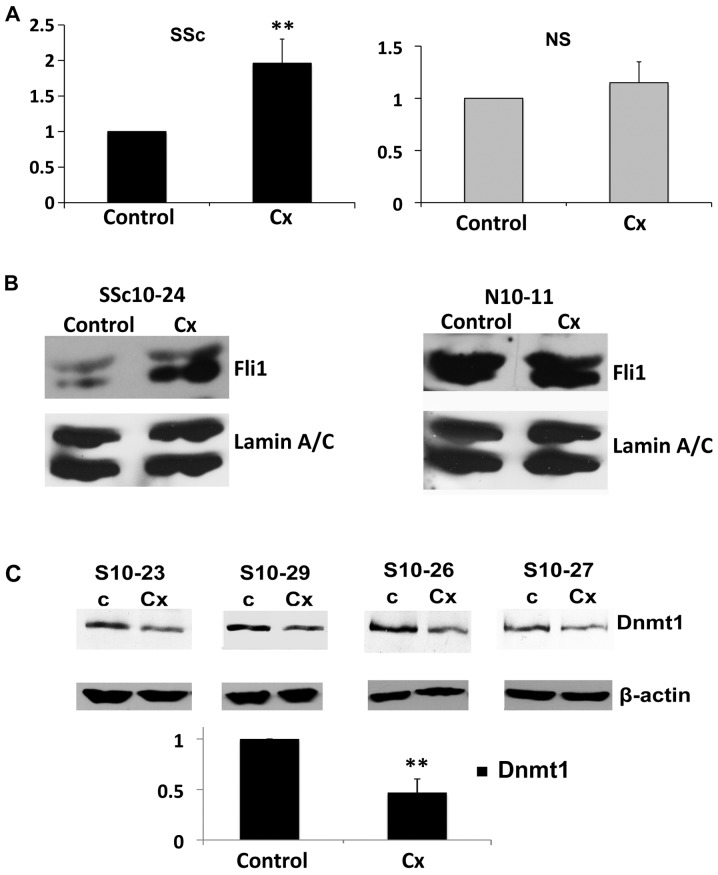
Following ciprofloxacin treatment Fli1 is increased and Dnmt1 is decreased in systemic sclerosis dermal fibroblasts. (A) Systemic sclerosis and healthy dermal fibroblasts were treated with 50 *μ*g/ml of ciprofloxacin for 48 h then mRNA levels of Fli1 were analyzed by RT-PCR (n=5). (B) Fli1 protein levels were examined by western blot analysis in both systemic sclerosis and healthy cells treated with 50 *μ*g/ml of ciprofloxacin for 96 h in nuclear lysates. Lamin A/C was used as a control for equal loading. (C) Protein levels of Dnmt1 were analyzed in 4 different systemic sclerosis cell lines 96 h after treatment with 50 *μ*g/ml ciprofloxacin. Bar graphs represent quantification compared to the control, arbitrarily set at 1. ^*^P≤0.05 and ^**^P≤0.01. Cx, ciprofloxacin; NS, healthy cells; SSc, systemic sclerosis.

We evaluated the effects of ciprofloxacin on Fli1 levels in normal and SSc dermal fibroblasts. Treatment of SSc fibroblasts with ciprofloxacin resulted in a statistically significant increase in Fli1 mRNA, leading to a 2-fold increase compared to untreated cells. However, when ciprofloxacin was added to the normal healthy fibroblasts, there was no effect on the mRNA levels of Fli1 (Fig. 4A). Essentially the same results were obtained at the protein levels, with a significant increase in Fli1 expression in SSc fibroblasts but with no change in the healthy cells after ciprofloxacin treatment (Fig. 4B).

Epigenetic repression of the Fli1 gene via methylation of cytosine nucleotides in the non-coding regions has been previously demonstrated to contribute to SSc fibrosis. Thus, a recent report demonstrated that the levels of epigenetic mediators, including the expression of methyltransferase Dnmt1, were altered in SSc fibroblasts (29). To examine whether epigenetic changes may be implicated in the ciprofloxacin-induced Fli1 upregulation in SSc dermal fibroblasts, four different SSc cell lines were treated with ciprofloxacin and the protein levels of Dnmt1 were then analyzed in cell lysates. In all cell lines there was a significant decrease in protein levels of Dnmt1 after ciprofloxacin treatment (>50%, P<0.0001; Fig. 4C).

### Ciprofloxacin has antifibrotic effects on lung fibroblasts isolated from SSc patients with ILD

**Figure 5:**
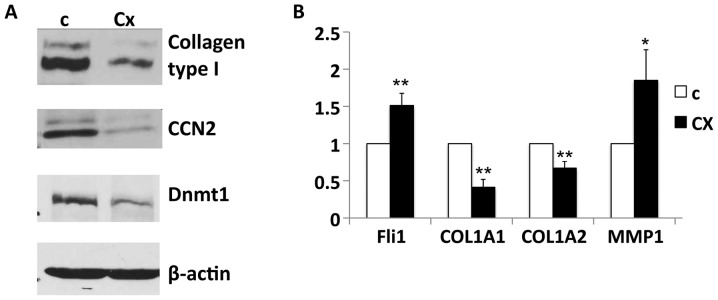
Ciprofloxacin has antifibrotic effects on systemic sclerosis lung fibroblasts. Quiescent lung fibroblasts isolated from systemic sclerosis patients with ILD were treated with 50 *μ*g/ml of ciprofloxacin then (A) protein and (B) mRNA levels of fibrotic molecules were analyzed using western blot analysis and RT-PCR. Bar graph represents the means ± SEM of 4 independent experiments in lung fibroblast cells, with ^*^P≤0.05 and ^**^P≤0.01. Cx, ciprofloxacin; SSc, systemic sclerosis; ILD, interstitial lung disease.

Since one of the major complications in SSc is pulmonary fibrosis, we next evaluated whether ciprofloxacin also reduces fibrotic markers in SSc lung fibroblasts isolated from patients with ILD. Ciprofloxacin potently downregulated collagen type I protein levels and decreased mRNA expression of both COL1A1 and COL1A2 chains (Fig. 5A and B). Similar to the results obtained in dermal fibroblasts, the levels of the profibrotic marker CCN2 were also downregulated after ciprofloxacin treatment, while MMP1 gene expression was enhanced. Furthermore, ciprofloxacin inhibited the levels of Dnmt1, while it upregulated Fli1 gene expression, thus suggesting that a similar mechanism accounts for the antifibrotic effects of ciprofloxacin in both dermal and lung fibroblasts isolated from SSc patients (Fig. 5). Based on these results, we concluded that ciprofloxacin has antifibrotic effects on human lung fibroblasts from SSc patients with ILD.

## Discussion

Skin and lung fibrosis in SSc are serious complications of the disease, which lack effective treatment. Intensive efforts have been made to discover new therapies that may ameliorate fibrosis in this disease. However, drugs that potently reduce fibrosis *in vitro* and in animal models have failed to provide reproducible antifibrotic effects in clinical trials for SSc patients. Intriguing preliminary data from a small clinical trial considering the effects of ciprofloxacin versus a placebo on skin fibrosis in SSc suggest that this antibiotic may be an effective treatment for skin fibrosis (12). In this study, we demonstrated that ciprofloxacin has dual antifibrotic effects on SSc dermal and lung fibroblasts by upregulating MMP1 and downregulating CCN2 and collagen type I levels. Furthermore, we provide evidence that Fli1 is upregulated after ciprofloxacin treatment only in SSc fibroblasts, but not in healthy cells, which were less responsive to the antifibrotic effects of antibiotic treatment. Additionally our study reveals that ciprofloxacin-induced MMP1 upregulation is mediated via the activation of the Erk1/2 pathway.

Tendinopathies, including tendon ruptures, have been described as rare but severe complications of ciprofloxacin treatment, with the main risk factors being old age, systemic steroid therapy, dialysis and strenuous physical activity. Although the exact pathologic mechanisms underlying this severe complication are poorly understood, several mechanisms have been proposed, including the upregulation of matrix metalloproteinases (MMPs) followed by type I collagen degradation, inhibition of cell proliferation by the downregulation of cyclin B and cyclin-dependent kinase 1 (Cdk1) and the inhibition of tenocyte migration by the downregulation of focal adhesion kinase phosphorylation (7,8,30–32). Thus, ciprofloxacin-induced collagen downregulation in tendon cells is the result of increased ECM degradation due to the upregulation of various MMPs. Fibrosis in SSc is the result of the uncontrolled deposition of ECM that is presumably due to increased synthesis and to decreased degradation of matricellular components. Ciprofloxacin may affect SSc fibrosis by regulating the aberrant expression of MMPs, leading to increased collagen turnover. MMP1 is the only enzyme capable of initiating the breakdown of interstitial collagens, including collagen type I, and published data indicates that this enzyme is downregulated in SSc cells (33). Our study demonstrates that, in addition to the effects on MMP1, ciprofloxacin treatment may directly block collagen synthesis in SSc fibroblasts, but not in healthy controls, by upregulating Fli1 levels.

The underexpression of Fli1 has been previously reported in fibrotic conditions such as SSc (15), cardiac fibrosis (34) and wound healing (35). Fli1 expression is markedly downregulated in lesional fibroblasts of patients with scleroderma, and a recent study has linked epigenetic regulation to the repression of the Fli1 gene in scleroderma skin *in vivo*. Thus, increased methylation of the Fli1 promoter was observed in SSc skin, while the authors did not discover any detectable methylation in the healthy control skin, suggesting Fli1 hypermethylation is specific for SSc fibroblasts (29). DNA methylation is an epigenetic regulation mechanism, resulting in transcriptional silencing of genes. This process is important for normal development but errors leading to promoter hypermethylation may play a major role in human disease. DNA methylation occurs by the addition of methyl groups to cytosine residues in DNA, catalyzed by a family of enzymes called DNA methyltransferases. Of the three types of Dnmt observed in mammalian cells, Dnmt1 was upregulated up to 3-fold in SSc fibroblasts, while Dnmt3a and Dnmt3b levels remained unchanged (29). In our study ciprofloxacin treatment potently decrease Dnmt1 levels in SSc dermal and lung fibroblasts, while upregulating Fli1 gene expression and downregulating COL1A1 and COL1A2 mRNA. Of note, ciprofloxacin had no effects on Fli1 levels in normal dermal fibroblasts, which also showed no reduction in collagen mRNA levels, suggesting that ciprofloxacin regulates Fli1 levels in SSc fibroblasts via an epigenetic mechanism that involves Dnmt1 downregulation.

Recent studies (19,25) from our laboratory demonstrated that the reduction of Fli1 levels in dermal fibroblasts results in an increased synthesis of type I collagen and CCN2 and a reduction in MMP1 production. In this study we revealed that ciprofloxacin treatment has opposite effects on collagen, CCN2 and MMP1. While transcriptional regulation of collagen type I genes in response to ciprofloxacin treatment is mainly controlled via Fli1, other factors may contribute to ciprofloxacin-induced MMP1 upregulation and CCN2 repression, since the mRNA levels of these genes are altered in both SSc and normal fibroblasts.

Erk1/2 activates the Ets1 transcription factor that in turn cooperates with the AP-1 proteins c-Fos and c-Jun in inducing the MMP1 promoter (19). Our present study demonstrates that ciprofloxacin induces Erk1/2 phosphorylation, and that the specific Erk1/2 inhibitor UO126 completely prevents ciprofloxacin-induced MMP1 upregulation, thus revealing that Erk1/2 is required for this process.

CCN2 and COMP are matricellular proteins that are over-expressed in SSc and are potently induced by TGFβ (36–38). Our previously published study showed that SSc fibroblasts have constitutive activation of Smad1 pathway, and that this pathway is directly involved in the upregulation of CCN2 gene expression (25). Additionally, Smad3 is required for the TGFβ induction of CCN2 (39). In our study, ciprofloxacin had no effects on TGFβ-induced phosphorylation of Smad1 or Smad3, thus suggesting that the downregulation of CCN2 and COMP in response to antibiotic treatment is independent of the TGFβ/Smad pathway. To the best of our knowledge, this is the first report to demonstrate that ciprofloxacin downregulates CCN2 and COMP in fibroblasts; however, further studies are required to elucidate the exact mechanism for these effects.

ILD in SSc is a potentially lethal complication with modest therapeutic options. Our study demonstrated that, similar to dermal fibroblasts, ciprofloxacin has antifibrotic effects on cultured SSc ILD lung fibroblasts, by upregulating Fli1 and MMP1 levels and downregulating collagen type I and CCN2 gene expression. The present data suggest that ciprofloxacin may also be an attractive antifibrotic therapy for SSc ILD.

**Figure 6:**
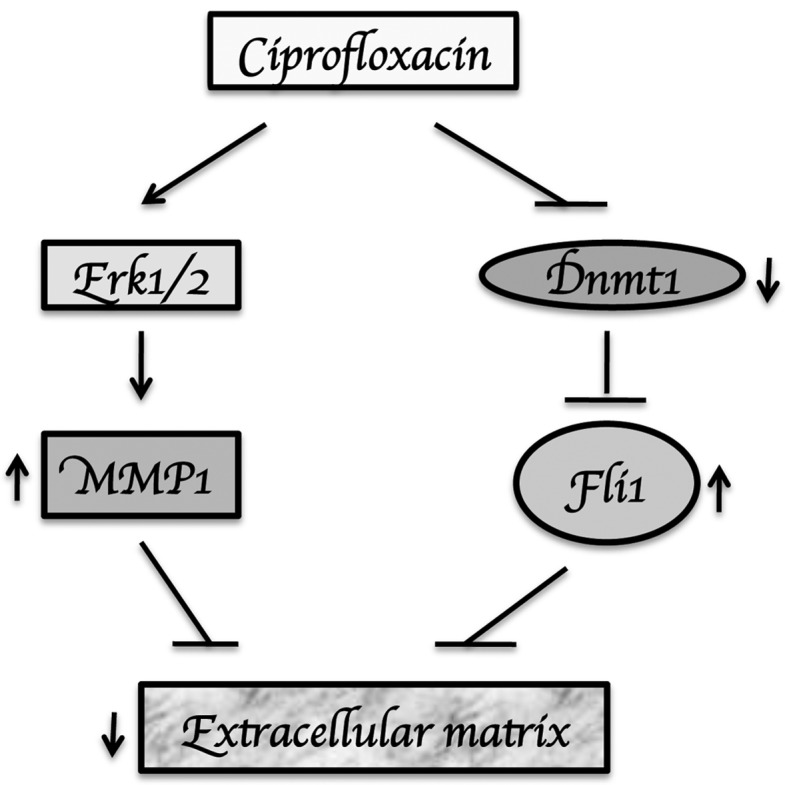
Schematic diagram showing the proposed mechanism of action for the antifibrotic effects of ciprofloxacin.

In summary, our study showed that ciprofloxacin has antifibrotic actions in SSc dermal and lung fibroblasts via downregulation of Dnmt1, upregulation of Fli1 and induction of MMP1 gene expression via an Erk1/2-dependent mechanism (Fig. 6). While these results provide evidence to support the use of ciprofloxacin in SSc, larger randomized clinical trials are warranted to confirm whether this may be a new treatment modality for SSc skin and lung fibrosis.

## References

[1] De Sarro A, De Sarro G (2001). Adverse reactions to fluoroquinolones. an overview on mechanistic aspects. Curr Med Chem.

[2] Lin HC, Yang YY, Tsai TH (2011). The relationship between endotoxemia and hepatic endocannabinoids in cirrhotic rats with portal hypertension. J Hepatol.

[3] Zhang M, Song G, Minuk GY (1996). Effects of hepatic stimulator substance, herbal medicine, selenium/vitamin E, and ciprofloxacin on cirrhosis in the rat. Gastroenterology.

[4] Reviglio VE, Hakim MA, Song JK, O’Brien TP (2003). Effect of topical fluoroquinolones on the expression of matrix metalloproteinases in the cornea. BMC Ophthalmol.

[5] Sharma C, Velpandian T, Baskar Singh S, Ranjan Biswas N, Bihari Vajpayee R, Ghose S (2011). Effect of fluoroquinolones on the expression of matrix metalloproteinase in debrided cornea of rats. Toxicol Mech Methods.

[6] Buyten J, Kaufman G, Ryan M (2007). Effects of ciprofloxacin/dexamethasone and ofloxacin on tympanic membrane perforation healing. Otol Neurotol.

[7] Tsai WC, Hsu CC, Chen CP (2011). Ciprofloxacin up-regulates tendon cells to express matrix metalloproteinase-2 with degradation of type I collagen. J Orthop Res.

[8] Corps AN, Harrall RL, Curry VA, Fenwick SA, Hazleman BL, Riley GP (2002). Ciprofloxacin enhances the stimulation of matrix metalloproteinase 3 expression by interleukin-1beta in human tendon-derived cells. A potential mechanism of fluoroquinolone-induced tendinopathy. Arthritis Rheum.

[9] Corps AN, Harrall RL, Curry VA, Hazleman BL, Riley GP (2005). Contrasting effects of fluoroquinolone antibiotics on the expression of the collagenases, matrix metalloproteinases (MMP)-1 and -13, in human tendon-derived cells. Rheumatology (Oxford).

[10] Sendzik J, Shakibaei M, Schafer-Korting M, Lode H, Stahlmann R (2010). Synergistic effects of dexamethasone and quinolones on human-derived tendon cells. Int J Antimicrob Agents.

[11] Goto K, Yabe K, Suzuki T, Takasuna K, Jindo T, Manabe S (2008). Gene expression profiles in the articular cartilage of juvenile rats receiving the quinolone antibacterial agent ofloxacin. Toxicology.

[12] Ruben EC, Manuel VR, Agustin OR, Huerta M, Antonio FM, Ivan DE (2010). Ciprofloxacin utility as antifibrotic in the skin of patients with scleroderma. J Dermatol.

[13] Truong AH, Ben-David Y (2000). The role of Fli-1 in normal cell function and malignant transformation. Oncogene.

[14] Czuwara-Ladykowska J, Shirasaki F, Jackers P, Watson DK, Trojanowska M (2001). Fli-1 inhibits collagen type I production in dermal fibroblasts via an Sp1-dependent pathway. J Biol Chem.

[15] Kubo M, Czuwara-Ladykowska J, Moussa O (2003). Persistent down-regulation of Fli1, a suppressor of collagen transcription, in fibrotic scleroderma skin. Am J Pathol.

[16] Asano Y, Markiewicz M, Kubo M, Szalai G, Watson DK, Trojanowska M (2009). Transcription factor Fli1 regulates collagen fibrillogenesis in mouse skin. Mol Cell Biol.

[17] Nakerakanti SS, Kapanadze B, Yamasaki M, Markiewicz M, Trojanowska M (2006). Fli1 and Ets1 have distinct roles in connective tissue growth factor/CCN2 gene regulation and induction of the profibrotic gene program. J Biol Chem.

[18] Haines P, Samuel GH, Cohen H, Trojanowska M, Bujor AM (2011). Caveolin-1 is a negative regulator of MMP-1 gene expression in human dermal fibroblasts via inhibition of Erk1/2/Ets1 signaling pathway. J Dermatol Sci.

[19] Bujor AM, Nakerakanti S, Morris E, Hant FN, Trojanowska M (2010). Akt inhibition up-regulates MMP1 through a CCN2-dependent pathway in human dermal fibroblasts. Exp Dermatol.

[20] Bu S, Yamanaka M, Pei H (2006). Dihydrosphingosine 1-phosphate stimulates MMP1 gene expression via activation of ERK1/2-Ets1 pathway in human fibroblasts. FASEB J.

[21] Jinnin M, Ihn H, Mimura Y, Asano Y, Yamane K, Tamaki K (2005). Matrix metalloproteinase-1 up-regulation by hepatocyte growth factor in human dermal fibroblasts via ERK signaling pathway involves Ets1 and Fli1. Nucleic Acids Res.

[22] Asano Y, Ihn H, Yamane K, Jinnin M, Mimura Y, Tamaki K (2005). Increased expression of integrin alpha(v)beta3 contributes to the establishment of autocrine TGF-beta signaling in scleroderma fibroblasts. J Immunol.

[23] Mimura Y, Ihn H, Jinnin M, Asano Y, Yamane K, Tamaki K (2006). Epidermal growth factor affects the synthesis and degradation of type I collagen in cultured human dermal fibroblasts. Matrix Biol.

[24] Jun JB, Kuechle M, Min J (2005). Scleroderma fibroblasts demonstrate enhanced activation of Akt (protein kinase B) *in situ*. J Invest Dermatol.

[25] Bujor AM, Asano Y, Haines P, Lafyatis R, Trojanowska M (2011). The c-Abl tyrosine kinase controls protein kinase Cdelta-induced Fli-1 phosphorylation in human dermal fibroblasts. Arthritis Rheum.

[26] Pannu J, Asano Y, Nakerakanti S (2008). Smad1 pathway is activated in systemic sclerosis fibroblasts and is targeted by imatinib mesylate. Arthritis Rheum.

[27] Mori Y, Chen SJ, Varga J (2003). Expression and regulation of intracellular SMAD signaling in scleroderma skin fibroblasts. Arthritis Rheum.

[28] Bujor AM, Pannu J, Bu S, Smith EA, Muise-Helmericks RC, Trojanowska M (2008). Akt blockade downregulates collagen and upregulates MMP1 in human dermal fibroblasts. J Invest Dermatol.

[29] Wang Y, Fan PS, Kahaleh B (2006). Association between enhanced type I collagen expression and epigenetic repression of the FLI1 gene in scleroderma fibroblasts. Arthritis Rheum.

[30] Williams RJ, Attia E, Wickiewicz TL, Hannafin JA (2000). The effect of ciprofloxacin on tendon, paratenon, and capsular fibroblast metabolism. Am J Sports Med.

[31] Tsai WC, Hsu CC, Tang FT, Wong AM, Chen YC, Pang JH (2008). Ciprofloxacin-mediated cell proliferation inhibition and G2/M cell cycle arrest in rat tendon cells. Arthritis Rheum.

[32] Tsai WC, Hsu CC, Chen HC (2009). Ciprofloxacin-mediated inhibition of tenocyte migration and down-regulation of focal adhesion kinase phosphorylation. Eur J Pharmacol.

[33] Takeda K, Hatamochi A, Ueki H, Nakata M, Oishi Y (1994). Decreased collagenase expression in cultured systemic sclerosis fibroblasts. J Invest Dermatol.

[34] Elkareh J, Periyasamy SM, Shidyak A (2009). Marinobufagenin induces increases in procollagen expression in a process involving protein kinase C and Fli-1: implications for uremic cardiomyopathy. Am J Physiol Renal Physiol.

[35] Sakthianandeswaren A, Curtis JM, Elso C (2010). Fine mapping of Leishmania major susceptibility Locus lmr2 and evidence of a role for Fli1 in disease and wound healing. Infect Immun.

[36] Abraham D (2008). Connective tissue growth factor: growth factor, matricellular organizer, fibrotic biomarker or molecular target for anti-fibrotic therapy in SSc?. Rheumatology (Oxford).

[37] Farina G, Lemaire R, Pancari P, Bayle J, Widom RL, Lafyatis R (2009). Cartilage oligomeric matrix protein expression in systemic sclerosis reveals heterogeneity of dermal fibroblast responses to transforming growth factor beta. Ann Rheum Dis.

[38] Farina G, Lafyatis D, Lemaire R, Lafyatis R (2010). A four-gene biomarker predicts skin disease in patients with diffuse cutaneous systemic sclerosis. Arthritis Rheum.

[39] Holmes A, Abraham DJ, Sa S, Shiwen X, Black CM, Leask A (2001). CTGF and SMADs, maintenance of scleroderma phenotype is independent of SMAD signaling. J Biol Chem.

